# Supplementary material for: Transmission of Raccoon-Passaged Chronic Wasting Disease Agent to White-Tailed Deer
Source: Viruses. 2022 Jul 20;14(7):1578. doi: 10.3390/v14071578 (PMC9320052; doi:10.3390/v14071578)

Name	Label	Rf	Mol. Wt.
Frame 1			
Chemi			
Marker lane 1			
Band 1		0.583	35
Band 2		0.705	25
Band 3		0.904	15
Rac CWD			
Band 1		0.613	30.27
Band 2		0.675	25.135
Band 3		0.762	20.887
WTD CWD			
Band 1		0.596	31.622
Band 2		0.662	26.216
Band 3		0.738	22.016
WTD 1542			
Band 1		0.603	31.081
Band 2		0.682	24.758
Band 3		0.748	21.532
WTD 1546			
Band 1		0.596	31.622
Band 2		0.669	25.676
Band 3		0.748	21.532
WTD 1555			
Band 1		0.596	31.622
Band 2		0.669	25.676
Band 3		0.745	21.694
WTD 1558			
Band 1		0.593	31.892
Band 2		0.662	26.216
Band 3		0.745	21.694
WTD 1561			
Band 1		0.586	32.432
Band 2		0.659	26.486
Band 3		0.732	22.339
WTD 1564			
Band 1		0.589	32.162
Band 2		0.659	26.486
Band 3		0.735	22.177
Marker lane 10			
Band 1		0.526	35
Band 2		0.649	25
Band 3		0.861	15

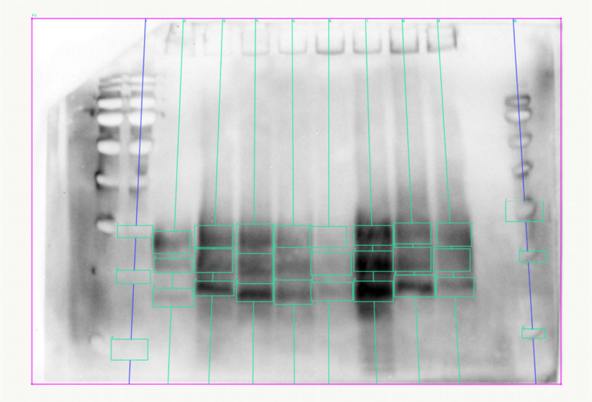

	Di	Mono	Un
Rac CWD	30.27	25.135	20.887
WTD CWD	31.622	26.216	22.016
WTD 1542	31.081	24.758	21.532
WTD 1546	31.622	25.676	21.532
WTD 1555	31.622	25.676	21.694
WTD 1558	31.892	26.216	21.694
WTD 1561	32.432	26.486	22.339
WTD 1564	32.162	26.486	22.177

	Di/Mo	Un
Rac CWD	1.204297	20.887
WTD CWD	1.20621	22.016
WTD 1542	1.255392	21.532
WTD 1546	1.231578	21.532
WTD 1555	1.231578	21.694
WTD 1558	1.216509	21.694
WTD 1561	1.224496	22.339
WTD 1564	1.214302	22.177

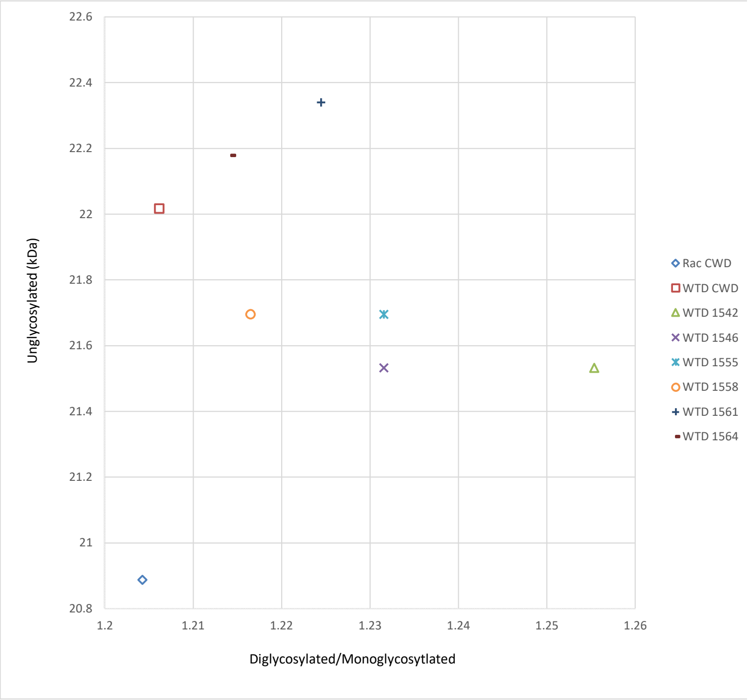

Supplement: Supplementary file 1 [file viruses-14-01578-s001.zip › Supplementary File 1.pdf]
