# Supplementary figures and images for: Transmission of Raccoon-Passaged Chronic Wasting Disease Agent to White-Tailed Deer
Source: Viruses. 2022 Jul 20;14(7):1578. doi: 10.3390/v14071578 (PMC9320052; doi:10.3390/v14071578)

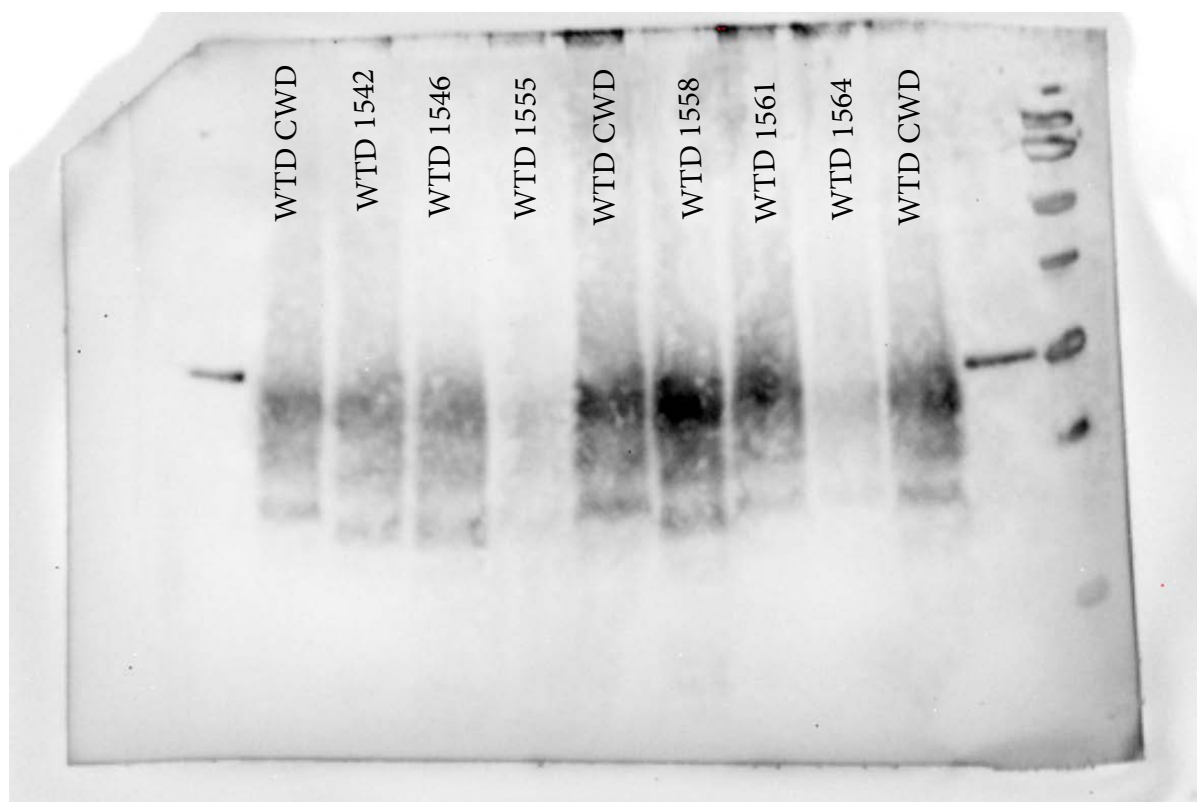

Supplement: Supplementary file 1 [file viruses-14-01578-s001.zip › Supplementary File 2.pdf]
